# Estimated Clinical and Economic Impact through Use of a Novel Blood Collection Device To Reduce Blood Culture Contamination in the Emergency Department: a Cost-Benefit Analysis

**DOI:** 10.1128/JCM.01015-18

**Published:** 2019-01-02

**Authors:** Erik Skoglund, Casey J. Dempsey, Hua Chen, Kevin W. Garey

**Affiliations:** a Department of Pharmacy Practice and Translational Research, University of Houston College of Pharmacy, Houston, Texas, USA; b Department of Pharmaceutical Health Outcomes and Policy, University of Houston College of Pharmacy, Houston, Texas, USA; Johns Hopkins University School of Medicine

**Keywords:** economic analysis, emergency department, health care-associated infections, microbiology, vancomycin

## Abstract

Blood culture contamination results in increased hospital costs and exposure to antimicrobials. We evaluated the potential clinical and economic benefits of an initial specimen diversion device (ISDD) when routinely utilized for blood culture collection in the emergency department (ED) of a quaternary care medical center. A decision analysis model was created to identify the cost benefit of the use of the ISDD device in the ED. Probabilistic costs were determined from the published literature and the direct observation of pharmacy/microbiology staff. The primary outcome was the expected per-patient cost savings (microbiology, pharmacy, and indirect hospital costs) with the routine use of an ISDD from a hospital perspective. The indirect costs included those related to an increased hospital length of stay, additional procedures, adverse drug reactions, and hospital-acquired infections. Models were created to represent hospitals that routinely or do not routinely use rapid diagnostic tests (RDT) on positive blood cultures. The routine implementation of ISDD for blood culture collection in the ED was cost beneficial compared to conventional blood culture collection methods. When implemented in a hospital utilizing RDT with a baseline contamination rate of 6%, ISDD use was associated with a cost savings of $272 (3%) per blood culture in terms of overall hospital costs and $28 (5.4%) in direct-only costs. The main drivers of cost were baseline contamination rates and the duration of antibiotics given to patients with negative blood cultures. These findings support the routine use of ISDD during blood culture collection in the ED as a cost-beneficial strategy to reduce the clinical and economic impact of blood culture contamination in terms of microbiology, pharmacy, and wider indirect hospital impacts.

## Abstract (precis)

Blood culture contamination results in increased hospital costs and exposure to antimicrobials. We evaluated the potential clinical and economic benefits of an initial specimen diversion device (ISDD) when routinely utilized for blood culture collection in the emergency department (ED) of a quaternary care medical center.

## INTRODUCTION

Blood culture contamination is a routine complication of patient care. The clinical uncertainty created by contaminated blood cultures decreases the diagnostic value of an initial report of positive growth and often results in detrimental downstream effects, such as increased diagnostic evaluations, unnecessary antibiotic exposure, increased hospital length of stay, increased risk of nosocomial infections, and increased strain on microbiology labs (1–6). The Clinical and Laboratory Standards Institute (CLSI) recommends an overall blood culture contamination rate of less than 3%; however, many institutions fail to meet this threshold, with rates of blood culture contamination ranging from 2% to greater than 10% using conventional techniques (2, 3, 6–8). Increased blood culture contamination rates have been observed in emergency departments (EDs) compared to those in general wards and intensive care units (ICUs) (9).

Several interventions have been utilized to decrease the risk of blood culture contamination, including sterile collection kits and a phlebotomy team (8–10). However, even with best practices, contaminants can represent up to half of all positive blood culture growth (7). Additionally, the cost benefit and sustainability of available interventions vary, preventing widespread adoption. A novel closed-system, mechanical blood culture diversion device that is preassembled and end-to-end sterile, Steripath (Magnolia Medical Technologies, Seattle, WA), which is also known in the literature as the initial specimen diversion device, or ISDD, was previously demonstrated to reduce the incidence of blood culture contamination by nearly 90% by diverting and sequestering the initial 1.5 to 2.0 ml of blood prior to culture bottle inoculation (4). However, an economic model to evaluate the cost benefit of ISDD implementation for routine blood culture collection does not exist. We sought to build a decision tree health care economic model to assess the cost benefit of routine use of an ISDD in a health system ED and to evaluate the downstream clinical and economic impacts of routine ISDD use in terms of microbiology, pharmacy, and indirect hospital costs.

## MATERIALS AND METHODS

### Decision model.

A decision analysis model was built using TreeAge software (TreeAge Software, Inc., Williamstown, MA). The structure of the decision tree was modified from a previously published model assessing the cost implications of blood culture contamination in the ED (10). This model was used to perform a cost-benefit analysis comparing the routine use of an ISDD for blood culture collection in the ED to the use of conventional practices without an ISDD for blood culture collection in the ED. Conventional methods were defined as collection by a nurse or phlebotomist via venipuncture with a clean, but nonsterile, technique using 2% chlorohexidine (CHG) in 70% isopropanol as the antiseptic or similar. The primary outcome was per-patient costs associated with ordering a blood culture in the ED, including microbiology, pharmacy, and indirect hospital expenditures. The tree model is shown in Fig. S1 in the supplemental material.

### Target population.

The target cohort for our decision tree model comprised all patients in the ED with an order for blood culture collection. Patients were excluded if they did not have two blood culture sets drawn as part of the initial order in the ED or if the blood culture yielded fungal growth (3). Culture results were adjudicated into three groups at the time of culture finalization for the purpose of evaluating costs: no bacterial growth, true bacteremia, or contaminated growth. Published studies used a definition of contamination to be blood culture growth due to skin-residing organisms (coagulase-negative staphylococci, *Propionibacterium* spp., *Micrococcus* spp., viridians group streptococci, *Corynebacterium* spp., or *Bacillus* spp.) if the growth was identified in ≤50% of available bottles as previously defined, generally considered one of two blood culture sets (2, 4).

### Data sources.

The information and model parameters for this study were primarily derived from published literature by using the primary data from the publication source via a systematic review of literature. When necessary, hospital charges were converted to hospital costs using a 0.3 cost-to-charge ratio (11). All costs were adjusted to 2017 U.S. dollars (USD) using the consumer price index (CPI) (12). In the absence of published data, information was obtained from institutional databases at an 884-bed quaternary care hospital with 78,000 annual ED visits located in the Texas Medical Center, Houston Texas. The collection of institutional data was approved by the institutional review board of the University of Houston and the participating hospital. The baseline estimates as well as the ranges included in the sensitivity analyses are presented in Table 1.

**TABLE 1:** Baseline decision tree parameters and ranges used in sensitivity analyses

Variable	Value at baseline	Sensitivity range	Reference(s) and/or source
Prevalence of true bacteremia (%)	7	7–7.5	2–4, 6, 8, 19
Rate of blood culture contamination at baseline (%)	6	2–10	2–4, 6, 8, 19
Rate of blood culture contamination with Steripath (%)	0.22	0–0.5	4
Probability of empirical antibiotics at culture collection (%)			
Negative or contaminated blood culture	71	64–78	Institutional database
True bacteremia	95	85–100	22, 23
Probability of stopping antibiotics by culture finalization (negative or contaminated culture) (%)	71	64–78	Institutional database
Administration of i.v. vancomycin (%)			
Negative blood culture	57	52–62	Institutional database
Contaminated blood culture	84	76–92	Institutional database
True bacteremia	66	60–72	Institutional database
Duration of inpatient antibiotics with negative blood culture (days)			
Empirical antibiotics, stopped by culture finalization	3	1–4	Institutional database
Empirical antibiotics, not stopped by culture finalization	9	7–13	Institutional database
No empirical antibiotics	0	0–5	Institutional database
Duration of inpatient antibiotics with contaminated culture (days)			
Empirical antibiotics, stopped by culture finalization	4	3–7	Institutional database
Empirical antibiotics, not stopped by culture finalization	10	7–13	Institutional database
No empirical antibiotics, stopped by culture finalization	1.5	1–3.5	Institutional database
No empirical antibiotics, not stopped by culture finalization	9	7–9	Institutional database
Duration of inpatient antibiotics with true bacteremia (days)	10	7–13	Institutional database
Hospital length of stay (days)			
Negative blood culture	5	3–9	1, Institutional database
Contaminated blood culture	7	4–11	1, Institutional database
True bacteremia	9	7–13	1, Institutional database
Costs ($)			
Blood culture collection and processing	36	20–56	6, 10, 15–17, 34
Organism identification and AST with RDT^*a*^	300	108–488	19–21, Institutional database
Organism identification and AST without RDT	104	80–200	6, 16–18, Institutional database
Daily antibiotic therapy (purchasing and labor)	75	50–80	12, 24
Serum vancomycin assay (laboratory)	68	63–77	35
Serum vancomycin assay (pharmacy)	41	28–55	Institutional database
Non-ICU (floor) (per day)	1,500	1,000–2,500	29
Follow-up tests and procedures	1,100	900–1,300	26–28
Hospital-acquired infection	5,000	2,500–10,000	30, 36, 37
Adverse drug reaction	150	25–600	31, 32

a AST, antimicrobial susceptibility testing; RDT, rapid diagnostic testing.

### Rate of blood culture contamination.

The incidence of bacterial growth from blood cultures drawn in the ED and the proportion of overall growth due to contamination were obtained from multiple observational studies. Published blood culture contamination rates using conventional collection methods have ranged from 2% to over 10% (1, 3, 4, 8, 9, 13, 14). The rates of overall bacterial growth and contamination with the use an ISDD were obtained from a controlled matched-pair trial by Rupp et al., in which a blood culture contamination rate of 0.22% was observed among 904 blood cultures (4). The investigators further demonstrated that the observed prevalence of true bacteremia was not affected by use of the ISDD (7.2%) compared to that with conventional techniques (7.6%, *P* = 0.41).

### Microbiology costs.

The cost of materials needed for the conventional collection of blood cultures was estimated on the basis of institutional costs and was corroborated by published data (10, 15). Opportunity labor costs were determined by the authors either by surveying or directly observing the microbiology staff over a period of 4 weeks at two sites: an 884-bed academic medical center and a 792-bed community hospital. The hourly wages for laboratory technicians were assigned according to the Bureau of Labor Statistics (BLS) occupational handbook (12). The initial instrument and material costs to process cultures were estimated on the basis of institutional costs and were corroborated by published data (6, 16–18). The costs of organism identification and antimicrobial susceptibility testing were determined separately for hospitals that utilize rapid diagnostic testing (RDT) (e.g., multiplex PCR, matrix-assisted laser desorption ionization–time of flight mass spectrometry [MALDI-TOF MS], and peptide nucleic acid probe fluorescence *in situ* hybridization [PNA-FISH]) and those that use conventional methods. The microbial identification and antimicrobial susceptibility testing costs were estimated as a composite that included the cost of reagents, supplies, and instrument acquisition divided by the expected number of samples to be processed over the life of the instrument (19–21). The cost estimates were calculated assuming routine identification and antimicrobial susceptibility testing was performed for all initial microbial growth isolated from blood samples.

### Antimicrobial administration and duration.

The duration of antibiotic therapy was estimated on the basis of the probability of two separate events: (i) receiving empirical therapy at the time of blood culture collection, and (ii) stopping therapy at the time of culture finalization. Patients were assumed to universally receive antibiotics at the initial report of unidentified bacterial growth from a blood culture if they were not started empirically. The duration of antibiotic therapy for patients with true bacteremia was not dependent on empirical therapy or de-escalation and was estimated on the basis of published observational data and the minimum recommended duration by the Infectious Diseases Society of America (22, 23). For other blood culture result categories, an institutional database was utilized to estimate the probability of starting or stopping inpatient antibiotics. A composite daily pharmacy cost of antibiotic provision was constructed utilizing institutional purchasing data for several broad-spectrum intravenous (i.v.) antibiotics at standard daily doses that are commonly given as empirical therapy in patients with suspected bloodstream infections: vancomycin ($20/day), cefepime ($25/day), meropenem ($30/day), linezolid ($80/day), and piperacillin-tazobactam ($20/day). Opportunity labor costs for the preparation and delivery of i.v. antibiotics were determined by the direct observation of pharmacy staff with hourly wages assigned according to the Bureau of Labor Statistics (BLS) occupational handbook (12). A point estimate of $75 was determined to represent the daily provision cost of antibiotics to a single patient, which included pharmacy purchasing and labor and was based on retrospective data that demonstrated patients with pathogenic or contaminated blood culture results were likely to concomitantly receive multiple antibiotics (24). Additional pharmacy labor costs were considered for therapeutic drug monitoring of vancomycin. Our model assumed that a patient receiving three or fewer days of vancomycin underwent one serum concentration assay, while patients receiving more than 3 days of vancomycin underwent two serum concentration assays (5, 24, 25). Pharmacist labor costs associated with assessing a serum vancomycin level were determined by the direct observation of staff and estimated to require 45 min to conduct an assessment and obtain a response.

### Indirect hospital costs.

The indirect costs included those related to an increased hospital length of stay, additional procedures, adverse drug reactions, and hospital-acquired infections. Published observational data were utilized to estimate the probabilistic cost of additional diagnostic or therapeutic interventions as a result of a positive blood culture, including central line placement/removal ($1,272), bone scan ($980), echocardiogram ($1,254), additional laboratory assays ($130), and diagnostic imaging ($1,700), with a final point estimate of $1,100 of additional diagnostic/procedural cost due to a positive blood culture (26–28). The costs associated with hospital length of stay were determined from published observational data and corroborated with institutional records of 3,325 unique patient encounters with blood cultures ordered in the ED (1). The occupation of a single-patient non-ICU hospital room was valued at $1,500 per day on the basis of published data and an institutional financial valuation (29). The risk of a hospital-acquired infection (HAI) was modeled using an incremental 1.37% risk per hospital day due to observational data demonstrating the majority of HAIs are experienced in the first 10 days of hospitalization, during which the incidence of HAIs increases linearly (30). The risk of experiencing an antibiotic-associated adverse drug reaction (ADR), such as nephrotoxicity or an infusion reaction, was estimated to increase incrementally at 6% per day of antibiotic therapy (31, 32).

### Cost-benefit analysis plan.

Expected value calculations were used to evaluate the cost benefit of the routine use of an ISDD to collect all blood cultures in the ED. Separate analyses were performed for hospitals that did and did not use RDT for organism identification and antimicrobial susceptibility testing. One-way sensitivity analyses were performed to assess the robustness of the results. The variables that were determined to have a significant effect on the outcome of our analysis were further subjected to two-way sensitivity analyses.

## RESULTS

### Clinical parameters associated with blood culture contamination and health care costs.

The data not available from our systematic review of the literature were obtained from our institutional database. Patients with contaminated blood cultures drawn in the ED were screened in a quaternary care hospital with a historical ED contamination rate of 6%. Between January and February 2017, 48 unique patient encounters were observed in which a contaminated blood culture was collected in the ED. To characterize the timing and duration of antibiotic therapy, this cohort was consecutively matched over the same period in a 1:1.5 ratio with patients whose ED blood cultures yielded no growth. Empiric therapy was initiated in 34 of 48 patients (71%) with contaminated cultures and in 50 of 70 patients (71%) whose cultures yielded no growth.

Of the 20 patients (29%) whose blood cultures yielded no growth and who were not started on empirical antibiotics on the day of culture collection, 14 (70%) were eventually started on antibiotics. Of these, antibiotics were stopped by the date of culture finalization in 10 patients (71%). Likewise, among patients whose blood cultures collected in the ED yielded no growth and did receive empirical therapy, 36 patients (72%) were discontinued from antibiotic therapy by the date of culture finalization. The durations of antibiotic therapy for these groups are displayed in Table 1.

Of the 14 patients (29%) with contaminated blood cultures who were not started on empirical antibiotics on the day of culture collection, 8 patients (57%) were eventually started on antibiotics. Of these, antibiotics were discontinued by the date of culture finalization in 6 patients (75%). Among patients with contaminated blood cultures who were started on empirical therapy, 23 patients (68%) were discontinued from antibiotic therapy by the date of culture finalization (Table 1).

Additional data from the same quaternary care hospital regarding hospital length of stay were extracted from 3,325 unique patient encounters in 2017 during which a blood culture was collected in the ED. Among these patients, the receipt of at least one dose of i.v. vancomycin was observed in 1,634 of 2,867 patients (57%) who had no bacterial growth identified from the initial ED blood culture, 136 of 206 patients (66%) who had true bacteremia, and 212 of 252 patients (84%) who had contaminated bacterial growth from the initial ED blood culture. The detection of bacterial growth from the initial ED blood culture was also associated with a longer hospital stay in this cohort. The median length of stay among patients with a contaminated blood culture (*n* = 253) was 7 days (interquartile range [IQR], 4 to 11 days), while the median length of stay among patients with a negative culture (*n* = 2,866) was 5 days (IQR, 3 to 9 days) (*P* < 0.0001).

### Costs due to blood culture contamination stratified by hospital use of RDT.

In hospitals that did not routinely use RDT, the overall hospital cost for patients with contaminated blood cultures was $12,824/patient, including costs from pharmacy ($422/patient) and microbiology ($275/patient) and indirect hospital costs ($12,126/patient). The overall costs increased in hospitals with routine use of RDT ($13,026) due to increased costs in microbiology ($477). The total cost per contaminated blood culture, total cost per negative blood culture, and the attributable cost per blood culture contamination are shown in Table 2.

**TABLE 2:** Distribution of component downstream costs stratified by result of initial blood culture collected in the ED

Category	Cost ($/culture)									
	Microbiology		Pharmacy	Hospital, indirect^*a*^					Total	
	With RDT^*b*^	Without RDT		LOS	ADRs	HAIs	Additional procedures	Total	With RDT	Without RDT
Contaminated blood culture	477	275	423	10,500	47	480	1,100	12,126	13,026	12,824
Negative blood culture	119	118	295	7,500	30	343	0	7,873	8,287	8,286
Attributable to blood culture contamination	358	158	127	3,000	16	137	1,100	4,253	4,739	4,538

a LOS, length of stay; ADR, adverse drug reaction; HAI, hospital acquired infection.

b RDT, rapid diagnostic testing.

### Base-case cost-benefit analysis of routine ISDD implementation.

Using baseline estimates from the quaternary care hospital and literature estimates, the routine implementation of an ISDD for blood culture collection in the ED was cost beneficial compared to conventional blood culture collection methods. Using a baseline contamination rate of 6%, the total expected cost of a blood culture patient episode was $8,893 using an ISDD and $9,165 with conventional methods in a hospital utilizing RDT, resulting in a cost savings of $272 (3.0% reduction in costs) per blood culture collection (Table 3). In a hospital not utilizing RDT, the total expected cost of a blood culture patient episode was $8,868 with an ISDD and $9,130 with conventional methods, resulting in a cost savings of $261 (2.9% reduction in costs) per blood culture collection. When considering only direct microbiology and pharmacy costs, the expected cost savings per blood culture collection were $28 (5.4% reduction in costs) in hospitals using RDT and $16 (3.4% reduction in costs) in hospitals not using RDT.

**TABLE 3:** Total estimated net cost savings per blood culture collection associated with routine Steripath implementation in the ED

Baseline blood culture contamination rate prior to Steripath implementation for routine blood culture collection (%)	Expected cost savings ($/culture)					
	Microbiology		Pharmacy	Hospital, indirect	Total	
	With RDT^*a*^	Without RDT			With RDT	Without RDT
2	6	3	2	74	83	79
3	10	4	3	117	130	124
4	13	6	4	160	178	170
6	21	9	7	244	272	261
8	28	12	10	330	367	352

a RDT, rapid diagnostic testing.

The cost-benefit analysis also showed that routine ISDD implementation was associated with reductions in antibiotic usage, adverse drug reactions, and hospital-acquired infections. ISDD implementation was associated with a 1.7% absolute reduction in the number of patients receiving at least one dose of vancomycin after blood culture collection in the ED. In a setting with 350 patient-unique blood cultures collected in the ED every month, ISDD implementation is associated with the complete avoidance of vancomycin administration in 6 additional patients per month.

### Sensitivity analyses.

**FIG 1:**
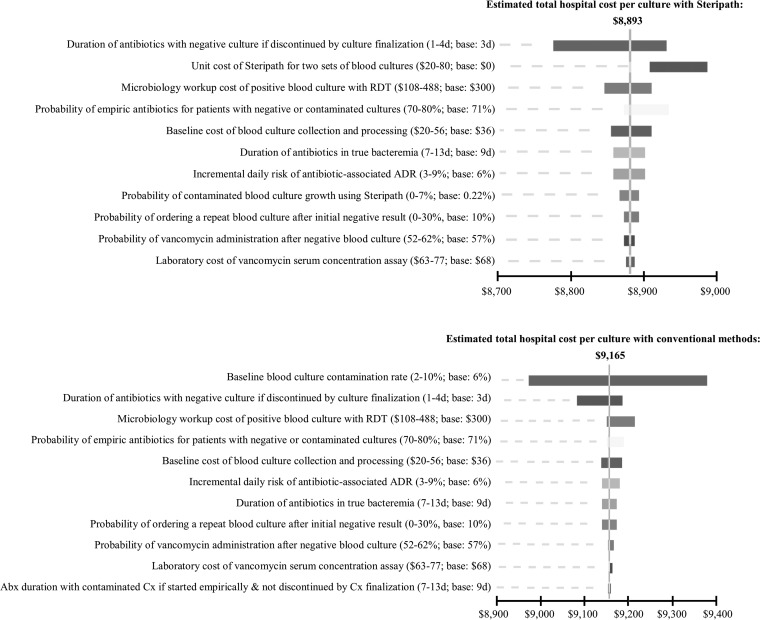
Tornado diagrams for estimated hospital cost per blood culture collection when routinely utilizing Steripath versus conventional methods in the ED. Data presented are from hospitals utilizing RDT for antimicrobial identification and susceptibility testing. Abx, antibiotic; Cx, culture.

**FIG 2:**
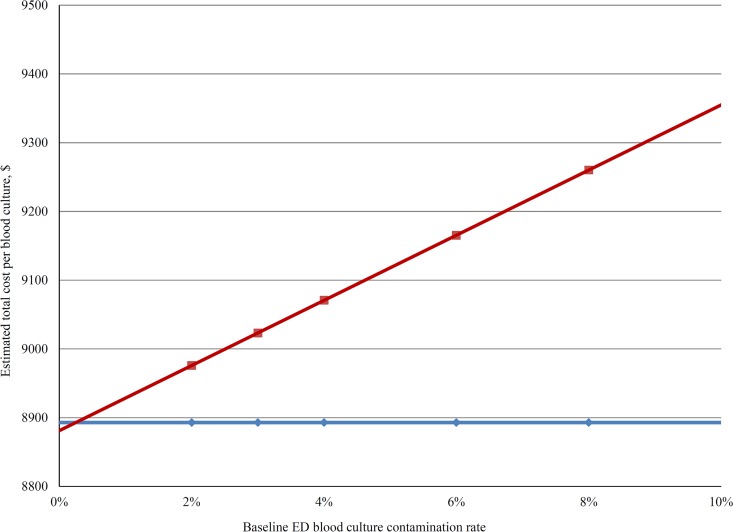
Total hospital costs associated with a blood culture collection using Steripath (blue line) versus conventional methods (red line) over a range of baseline blood culture contamination rates. Data presented are from hospitals utilizing RDT for antimicrobial identification and susceptibility testing. Estimated cost differential shown does not include the cost of Steripath units.

The results of the sensitivity analysis confirmed the robustness of the model to a range of base-case parameter values. The variables that most influenced the model in hospitals that use conventional collection techniques versus those with routine use of the ISDD are shown in Fig. 1. To perform a more conservative evaluation of the cost benefit of routine ISDD implementation in the ED, one- and two-way sensitivity analyses were also performed considering only direct purchasing and labor costs within the pharmacy and microbiology departments. Under these conditions, the threshold values for the unit cost of an ISDD at which the strategy of routine ISDD use was equal in direct costs to the conventional blood culture collection strategy were $28 with RDT and $16 without RDT. When considering the total hospital expenditure, including indirect costs, the threshold values for the unit cost of an ISDD at which the strategy of routine ISDD use was equal to the conventional blood culture collection strategy were $272 with RDT and $261 without RDT. The total hospital costs associated with blood culture collection using Steripath versus conventional methods over a range of baseline blood culture contamination rates are shown in Fig. 2.

**FIG 3:**
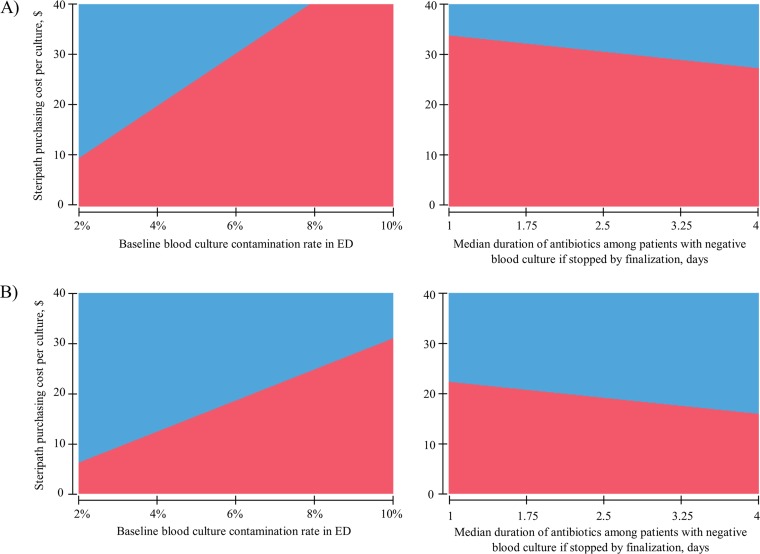
Two-way sensitivity analyses for direct (microbiology and pharmacy) costs per blood culture collection in the ED associated with two blood culture collection strategies (break-even analysis). (A) Hospitals using RDT. (B) Hospitals utilizing traditional microbiology identification and susceptibility techniques. Blue shading represents conventional blood culture collection preferred, and red shading represents routine Steripath implementation preferred.

A two-way sensitivity analysis of the expected per culture direct (pharmacy and microbiology) cost of routine ISDD use versus that for conventional methods of blood culture collection in a hospital with RDT demonstrated that the use of an ISDD was the least costly strategy at an ISDD unit cost of $30 over a range of baseline blood culture contamination rates above 6% (Fig. 3). Likewise, the use of an ISDD was the least costly strategy in hospitals using RDT at a unit cost of $30 when the median duration of antibiotic therapy was less than 3 days among patients with negative blood cultures whose therapy was discontinued by culture finalization.

## DISCUSSION

Blood culture contamination in emergency departments increases hospital costs and affects patient outcomes. In this cost-benefit study, the routine use of an ISDD was a cost-saving strategy compared to conventional methods over a range of baseline variables. The results of this study demonstrate that the use of an ISDD to decrease blood culture contamination rates also decreases associated hospital costs from multiple hospital departments. The strengths of the study include the use of a systematic literature review supplemented with real-world observation and databases to provide estimates, dual analyses based on microbiology use of rapid diagnostic tests, and the identification of cost drivers that are affected by blood culture contamination.

The clinical utility of the Steripath ISDD was demonstrated using a cohort of phlebotomist-collected blood cultures (4). In this study, blood culture sets were collected with and without an ISDD. The rate of blood culture contamination without using an ISDD was 1.78%, which decreased to 0.22% with use of ISDD; an 87.6% reduction. While no published studies have evaluated the economic impact of routine implementation of ISDDs, the cost-effectiveness of other interventions designed to decrease the rate of blood culture contamination has been studied. A decision tree cost analysis with a baseline contamination rate of 4.34% demonstrated that the use of sterile kits or phlebotomy teams for blood culture collection was associated with a net hospital cost savings compared to usual practices (10). Our results showed a similar cost-effectiveness benefit in addition to a more granular analysis of areas where cost savings are observed. Sustainability and work flow practicality associated with dedicated phlebotomy teams in a busy ED were a limitation of the previous study. Interventions that rely on new methodology versus constant staff education or the presence of specialists may also be more sustainable over time (10, 33). Other antimicrobial stewardship benefits associated with the ISDD device should be studied in the future.

While this study was designed within a framework that can be generalized to a wide range of institutions, (e.g., those with or without access to RDT), there are important considerations to note. This study assumed that all bacterial growth identified from a blood culture was subjected to full microbiologic identification and susceptibility testing; however, not all institutions are likely to subject every organism identified as a potential skin contaminant to full antimicrobial susceptibility testing. Furthermore, this study utilized an aggregated composite estimate for RDT comprising a range of available laboratory instruments with various acquisition and operating costs. We accounted for these differences in clinical practice by performing separate analyses for hospitals that do or do not routinely perform RDT as well as a wide range of possibilities in the sensitivity analysis. Hospitals vary widely in their use of RDT, and further refinements to the model can be undertaken for differing scenarios. We did not account for any wastage of the ISDD or additional time needed to use or dispose of the device. We modeled the effect of the ISDD in the ED, an area of health care with high rates of contamination. Models that predict economic benefit in other areas of the health care continuum will be needed. The cost-benefit analysis in this study was of predicted probabilities only, and further real-world clinical trial evidence will be required to confirm these results. Additional limitations to this study to consider include the heterogeneous nature of the data used to compile point estimates; however, data obtained from disparate sources were corroborated or reconciled by review of institutional databases when available.

### Conclusion.

These findings support the routine use of an ISDD for the collection of blood cultures in the ED as a cost-beneficial strategy to reduce the clinical and economic effect of blood culture contamination in terms of microbiology, pharmacy, and wider indirect hospital implications.

## Supplementary Material

Supplemental file 1
