# Supplementary figures and images for: Estimated Clinical and Economic Impact through Use of a Novel Blood Collection Device To Reduce Blood Culture Contamination in the Emergency Department: a Cost-Benefit Analysis
Source: J Clin Microbiol. 2019 Jan 2;57(1):e01015-18. doi: 10.1128/JCM.01015-18 (PMC6322461; doi:10.1128/JCM.01015-18)

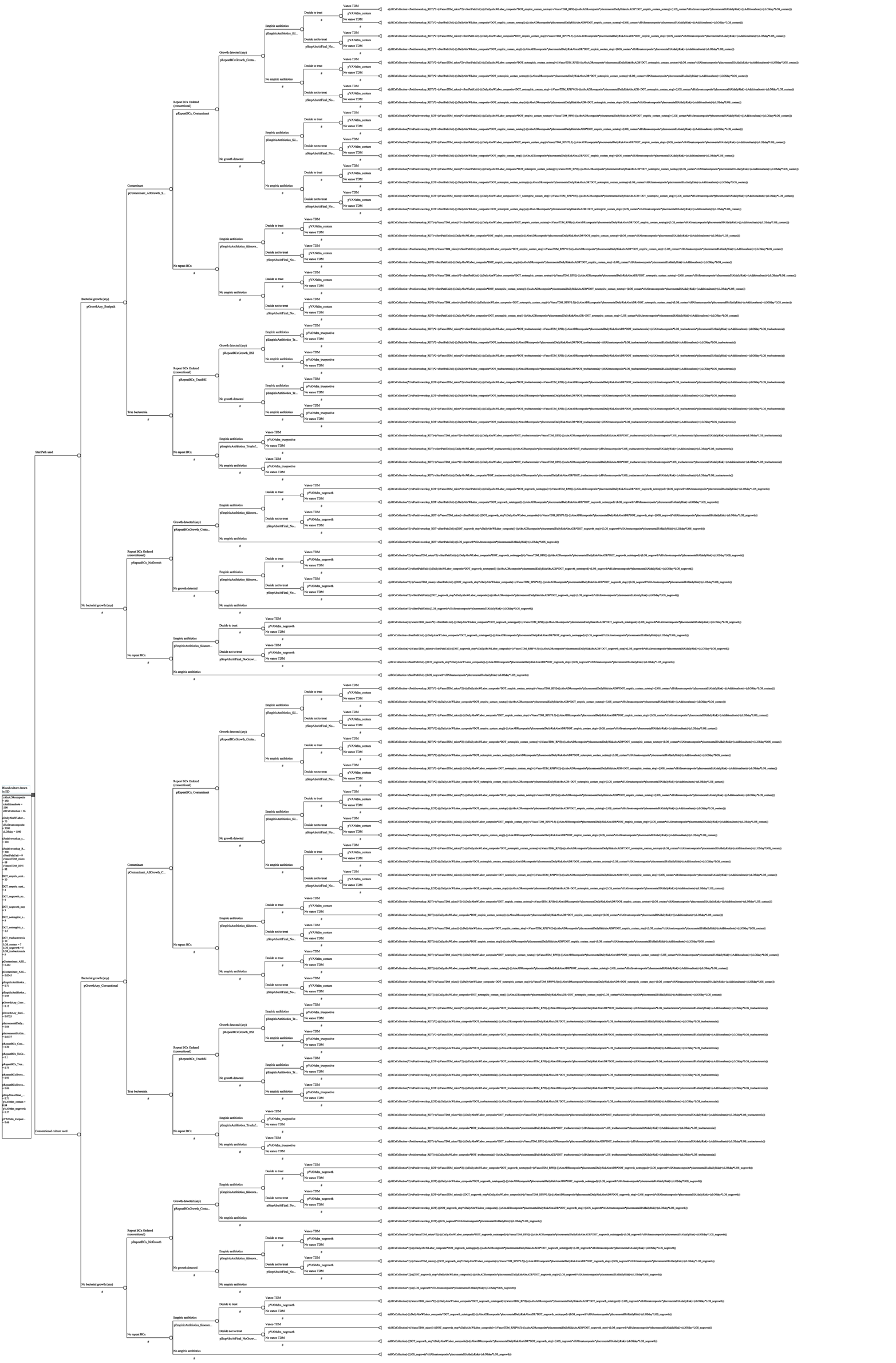

Supplement: Supplemental file 1 [file c98889028bee6d29ee27d028b2eae693_JCM.01015-18-s0001.pdf]
